# Analysis of risk factors for changes of left ventricular function indexes in Chinese patients with gout by echocardiography

**DOI:** 10.3389/fphys.2023.1280178

**Published:** 2023-11-23

**Authors:** Wantai Dang, Danling Luo, Jing Hu, Hui Luo, Xiaohui Xu, Jian Liu

**Affiliations:** ^1^ Department of Rheumatology and Immunology, Clinical Medical College, The First Affiliated Hospital of Chengdu Medical College, Chengdu, China; ^2^ Department of Ultrasound, Clinical Medical College, The First Affiliated Hospital of Chengdu Medical College, Chengdu, China

**Keywords:** left ventricular function, gout, echocardiography, uric acid, risk factors

## Abstract

**Background:** Echocardiographic data investigating the association between left ventricular (LV) function and gout is still limited.

**Purpose:** To analyze the association of echocardiographic parameters based on two-dimentional speckle tracking analysis with clinically related indicators in patients with gout, and to provide a clinical basis for the early diagnosis and treatment of cardiovascular disease in patients with gout.

**Methods:** This study collected gout patients who visited the outpatient and inpatient departments of the first affiliated hospital of chengdu medical college from November 2019 to December 2020. Spearman correlation test was performed to analyze the correlation coefficients between the laboratorial indicators with echocardiographic parameters. And the logistic regression analysis was performed to evaluate the independent effects.

**Results:** The results of multivariate logistic regression showed that fasting plasma glucose (FPG) was a risk factor for the decrease in absolute value of global longitudinal strain [GLS (OR = 2.34; 95% CI, 1.01–5.39; *p* = 0.04)], Urea was a risk factor for absolute reduction in GCS (OR = 1.40; 95% CI, 1.07–1.85; *p* = 0.02), age (OR = 1.09, 95% CI, 1.04–1.16; *p* = 0.001), and hypertension (OR = 8.35; 95% CI, 1.83–38.02; *p* = 0.006) were risk factors for increased E/Em. High urea levels were significantly related with high risks of LVH (OR = 1.59, 95% CI, 1.04–2.43; *p* = 0.03) and enlargement of LAVI (OR = 1.68, 95% CI, 1.01–2.80; *p* = 0.04).

**Conclusion:** Our study found that elevated urea and FPG were risk factors for subclinical LV myocardial dysfunction in patients with gout, which might provide a theoretical basis for the early diagnosis and treatment of heart disease in clinical practice.

## 1 Introduction

Gout is the most common form of inflammatory arthritis characterized by deposition of monosodium urate monohydrate (MSU) in synovial fluid and other tissues, with a prevalence of 1.1% in mainland China, and 6.8% of the population worldwide (Neogi, 2011; Liu et al., 2015; Dehlin et al., 2020). Previous studies has reported that cardiovascular events are positively associated with gout. The relationship between SUA and cardiovascular events is also complex and requires further study. To better understand the relationship between SUA and cardiovascular events and to develop effective prevention and treatment strategies, we must take into account the widespread (and often underestimated) conditions such as incorrect lifestyle, obesity, and insulin resistance that can harm SUA levels while increasing cardiometabolic risk (Maloberti et al., 2023). A recent observational evidence demonstrated that patients with gout were found to have a higher burden of co-morbidities and a higher rate of all-cause hospitalization compared to patients without gout (Carnicelli et al., 2020). Another study showed that the frequency of gout attacks was associated with an odds ratio (OR) of 1.18 for myocardial infarction (Chen et al., 2007). In patients with gout, hyperuricemia mainly upregulates xanthine oxidase activity leading to increased oxidative stress and monosodium urate crystal-mediated inflammation that affects the pathophysiological changes of cardiovascular disease (Eisen et al., 2013; Pan et al., 2014a). But study have shown that hyperuricemia is not the only risk factor for left ventricular (LV) dysfunction in gout patients (Lin et al., 2015).

In recent years, echocardiographic techniques has been rapidly developed as a novel auxiliary technique for assessing the structure and function of the heart. One of which is speckle tracking, which is principally based on the analysis of speckles during the cardiac cycle (Mondillo et al., 2011). This technique can be used to study global myocardial deformation via global longitudinal strain (GLS) and global circumferential strain (GCS) assessment (Reant et al., 2010; Zhang et al., 2018). This method trackes the scattered spots evenly distributed in the myocardium in the two-dimensional gray-scale image and the movement of its geometric position frame by frame, and applys spatial and temporal image processing algorithms to obtain tissue motion information, thereby obtaining multiple motion parameters in longitudinal, radial and circumferential directions. Moreover, it overcomes the angle dependence and can more accurately reflect the myocardial movement and evaluate the myocardial strain capacity (Langeland et al., 2005). Therefore, it can serve as a powerful quantitative tool to assess global and regional LV function. In addition, measurement of this index is now possible on-line during the ultrasound examination, with no requirement for post-processing.

Although study has not found significant change on LV structure and systolic function in gout patients, there were evidences suggested that the severity of gout can lead to LV diastolic dysfunction and subclinical systolic dysfunction (Pan et al., 2014b; Naghavi et al., 2016). In addition, echocardiographic data investigating the association between LV function and gout is still limited. Speckle-tracking echocardiography–derived measurements have been validated against sonomicrometry and tagged MRI, showing high feasibility and reproducibility (Amundsen et al., 2006). The purpose of this study was to analyze the association of echocardiographic parameters based on two-dimentional speckle tracking analysis with clinically related indicators in patients with gout, and to provide a clinical basis for the early diagnosis and treatment of cardiovascular disease in patients with gout.

## 2 Methods

### 2.1 Subjects and study design

This study collected gout patients who visited the outpatient and inpatient departments of the first affiliated hospital of chengdu medical college from November 2019 to December 2020. The inclusion criteria: 1) diagnosis of gout based on the definition of the American College of Rheumatology (ACR) and European League Against Rheumatism (EULAR); 2) have not received any related therapies (within 3 months) to reduce uric acid, blood pressure, and glucose; 3) agree to sign the informed consent. The exclusion criteria were: 1) patients with secondary gout caused by malignant tumor, radiotherapy, chemotherapy, or blood system diseases; 2) with previous valvular heart disease, ischemic cardiomyopathy, dilated or hypertrophic cardiomyopathy, coronary artery disease, chronic lung disease, or severe anemia; 3) with arrhythmia, pacemaker implantation, or previous history of cardiac surgery; 4) with autoimmune diseases such as rheumatoid arthritis and ankylosing spondyloarthritis (excluding immune-related indicators such as rheumatoid factor and anti-citrulline peptide autoantibodies). Fifty-one healthy adults were recruited from the same hospital’s health management center as a control group, screened through history, physical examination, and laboratory tests to exclude any individuals with conditions such as hypertension and diabetes, and echocardiography was performed on them. This study was approved by the Institutional Review Board of our hospital and written informed consent was obtained from all subjects participating in the study.

### 2.2 Echocardiography

**FIGURE 1:**
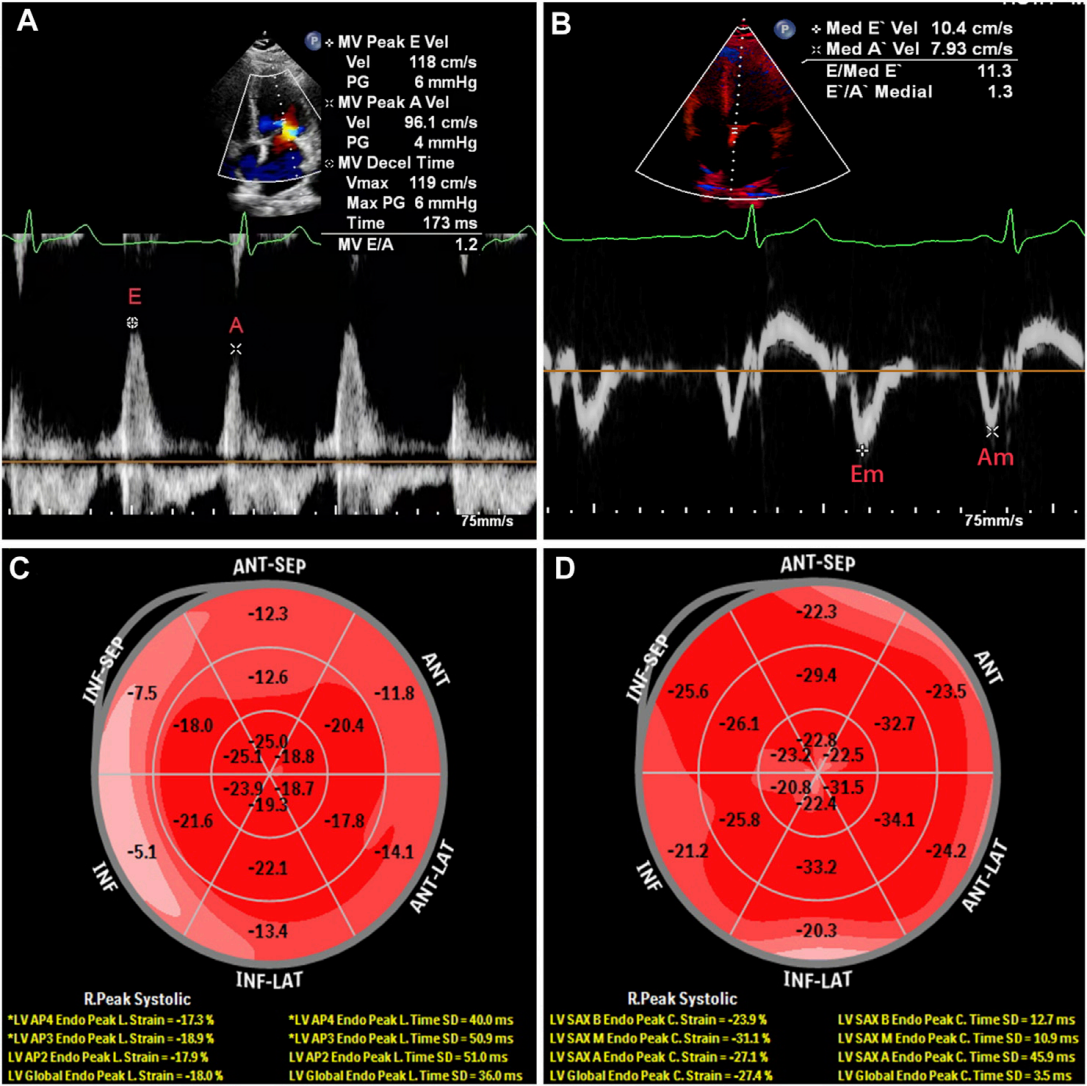
Echocardiography of a 34-year-old male patient diagnosed with gout without secondary hypertension and diabetes. **(A)**, early diastolic peak of mitral valve orifice; **(B)**, the peak early diastolic mitral annular velocity; **(C)**, global longitudinal strain (GLS); **(D)**, global circumferential strain (GCS).

All subjects received two-dimensional echocardiographic examinations at rest in the left lateral decubitus position using a EPIQ 7C color Doppler ultrasound diagnostic apparatus (1–5 MHz, Philips, Washington, United States) and S5-1 broadband phased array transducer. According to the current guidelines of the American Society of Echocardiography (Mitchell et al., 2019), early diastolic peak of mitral valve orifice (E) were measured at the mitral valve tip by pulse-wave tissue Doppler imaging (TDI) in the apical four-chamber view (Figure 1A). The peak early diastolic mitral annular velocity (Em) were obtained at the mitral valve tip by pulse-wave TDI at the septal site of the mitral annulus (Figure 1B). The E/Em ratio was used as an index of LV filling pressure (Nagueh et al., 2016; Oh et al., 2020). The left ventricular end-diastolic diameter (LVDd), end-diastolic septal thickness (IVST), and end-diastolic left ventricular posterior wall thickness (PWT) were measured for 3 continuously cardiac cycles, and calculated the average value of 3 measures. The left ventricular mass (LVM) was calculated according to the formula recommended by the American Society of Echocardiography (ASE) (Lang, 2016): LVM(g) = 0.8 × 1.04×[(IVST + PWT + LVDd)^3^-LVDd^3^] + 0.6. After standardized the LVM by body surface area (BSA), we calculated the left ventricular mass index (LVMI) as LVMI(g/m^2^) = LVMI/BSA. Left ventricular hypertrophy (LVH) was diagnosed as LVMI>95 g/m^2^ in females and LVMI>115 g/m^2^ in males (Lang, 2016). The left atrial volume index (LAVI) was calcualted and left ventricular ejection fraction (LVEF), which represented the LV systolic function, was calculated. Two-dimensional dynamic images of three apical views (four-, two- and three-chamber) were recorded, and all echocardiographic measures were obtained through consecutive three heart cycles. Speckle-tracking analysis was performed using Qlab software. Two experienced ultrasound physicians independently measured the GLS and GCS, and the intra-group correlation coefficients were > 0.90 for both GLS and GCS which indicated for a good consistency. GLS and GCS values were calculated respectively as the means of the global longitudinal strains and global circumferential strain of each apical view (Figures 1C, D). LAVI enlargement was defined as LAVI > 34 mL/m^2^. The absolute decrease in GLS was defined as GLS > −20%, and the absolute decrease in GCS was defined as GCS > −30%. The increase in E/Em was defined as E/Em > 14 (Lang et al., 2015).

### 2.3 Laboratory

3 mL of fasting blood was collected from the patient (no food and drink for at least 8 h overnight) in the morning, and examined with an automatic hematology analyzer (Japan, SYSMEX, xn-9000) on the day of echocardiographic examination. The erythrocyte sedimentation rate was analyzed (Italy, VITAL diagnostics, MONITOR-100). Laboratorial indicators related to renal function, lipid metabolism, and blood glucose were measured by an automatic biochemical analyzer (Japan, Hitachi Limited, 7600). The data of red blood cell (RBC), hemoglobin (HGB), platelets (PLT), neutrophils (NE), lymphocytes (LY), Urea, cystatin C (Cys-C), β2-microglobulin (β2-MG), total protein (TP), albumin (ALB), albumin/globulin (A/G), triglyceride (TG), total cholesterol (TC), low-density lipoprotein cholesterol (LDL-C), apolipoprotein A-I (ApoA-I), apolipoprotein B (ApoB), lipoprotein (a) (Lp(a)), fasting plasma glucose (FPG), glycated hemoglobin (HBA1C), average blood glucose (AG), C-reactive protein (CRP), and erythrocyte sedimentation rate (ESR) were recorded for analysis.

### 2.4 Statistical analysis

All the data were analyzed by SPSS Software v22.0 (IBM Corporation, Armonk, NY, United States). Normally distributed data was presented in mean ± standard deviation. We perform a normality test on the selected variable by performing a probe analysis and checking the normality option. According to the Kolmogorov-Smirnov and Shapiro-Wilk tests, GLS, GCS, E/Em, LVEF, LVMI and LAVI all agree with the hypothesis of normal distribution. The intra-class correlation coefficient (ICC) was calculated to evaluate the consistency of the GLS and GCS measurements between the two ultrasound physicians (ICC > 0.75 indicates a good consistency). Spearman correlation test was utilized to examine the correlation coefficients between laboratorial indicators and echocardiographic parameters. Furthermore, logistic regression analysis was applied to assess the independent effects of the laboratorial indicators on changes in GLS, GCS, LVMI, LAVI and E/Em, respectively, while controlling for other covariates. The variable selection method for building the regression models was a stepwise approach, which was performed after the initial correlation analysis. A *p*-value < 0.05 was considered statistical significant.

## 3 Results

A total of 227 cases including 215 males (94.7%) and 12 females (5.3%) were included in the study, of which, 40 patients cannot obtain GLS and GCS due to poor image resolution. The average age of the included patients were 45.71 ± 15.33 years old. The baseline characteristics of included patients were summarized in Table 1. Among these patients, 164 had decreased GLS, 96 had decreased GCS, 20 had increased E/Em, 16 had LVH, and 16 had increased LAVI. Table 2 shows the statistical results between the complete sample and the reduced sample using GLS and GCS. The results show that there are no significant differences in Age, BMI and Course of disease between the two groups (*p* > 0.05).

**TABLE 1:** Characteristics of gout patients and healthy controls, and echocardiographic parameters.

Characteristics	Gout		Normal	
	N (%)	Mean ± SD	N (%)	Mean ± SD
Age(year)	227	45.71 ± 15.33	51	48.33 ± 14.98
Gender				
Male	215 (94.7%)	-	47(92.2%)	-
Female	12 (5.3%)	-	4(7.8%)	-
BMI (kg/m^2^)	227	26.01 ± 3.77	51	24.21 ± 2.68
Course of disease(year)	227	4.70 ± 5.53	-	-
Hypertension	43(19%)	-	-	-
Diabetes	28(12%)	-	-	-
Tophi	104(46%)	-	-	-
Echocardiographic parameter				
GLS (%)	187	−17.53 ± 2.31	51	−19.70 ± 2.33
GCS (%)	187	−30.16 ± 5.01	51	−32.59 ± 3.75
LVEF (%)	227	62.96 ± 4.90	51	63.47 ± 3.28
E/Em	227	10.25 ± 3.16	51	6.19 ± 1.10
LVMI(g/m^2^)	227	81.29 ± 19.86	51	70.73 ± 15.54
LAVI(mL/m^2^)	227	24.10 ± 7.09	51	24.67 ± 4.86

Note: BMI: body mass index; GLS: global longitudinal strain; GCS: global circumferential strain; LVEF: left ventricular ejection fraction; E: diastolic peak early transmission flow velocity; Em: Peak early diastolic mitral annulus velocity; LVMI: left ventricular myocardial mass index; LAVI: left atrial volume index.

**TABLE 2:** Comparison of basic characteristics of the complete sample and the reduced sample using GLS and GCS.

Characteristics	Complete sample	Reduced sample	t	*P*
Age(year)	45.71 ± 15.33	46.31 ± 15.39	0.39	0.69
BMI (kg/m2)	26.01 ± 3.77	25.92 ± 3.59	0.27	0.79
Course of disease(year)	4.70 ± 5.53	4.64 ± 5.39	0.12	0.91

Compared with the normal group, the absolute values of GLS and GCS in the gout group were lower, E/Em and LVMI were larger, while LVEF and LAVI were not different between the two groups (The *p*-values of GLS, E/Em and LVMI were all < 0.001, and the *p*-values of GCS were 0.001. The *p*-values of LVEF and LAVI were all > 0.05).

Table 3 presents the results of a univariate analysis of variables with statistical differences and variables of clinical significance, where GLS, GCS, E/Em, LVH and LAVI were used as dependent variables, while laboratory indicators served as independent variables. This analysis aimed to examine the individual associations between each laboratory indicator and the dependent variables. Table 4, on the other hand, summarizes the results of further multivariate logistic regression analysis. In this analysis, we included the laboratory indicators that were found to be significantly associated with the dependent variables in the univariate analysis. The purpose of multivariate regression was to evaluate the combined effects of these laboratory indicators on GLS, GCS, and E/Em, while adjusting for potential confounding factors. By performing multivariate logistic regression analysis, we were able to assess the independent contributions of the laboratory indicators, while controlling for other variables, and obtain more comprehensive and accurate conclusions regarding their relationships with GLS, GCS, and E/Em, LVH and LAVI. This approach allowed us to account for potential confounders and better understand the association between the laboratory indicators and the dependent variables of interest. The results showed that FPG was a risk factor for the decrease in absolute value of GLS (OR = 2.34; 95% CI, 1.01–5.39; *p* = 0.04); urea was a risk factor of absolute reduction in GCS (OR = 1.40; 95% CI, 1.07–1.85; *p* = 0.02); age (OR = 1.09, 95% CI, 1.04–1.16; *p* = 0.001), and hypertension (OR = 8.35; 95% CI, 1.83–38.02; *p* = 0.006) were risk factors for increased E/Em. High urea levels were significantly related with high risks of LVH (OR = 1.59, 95% CI, 1.04–2.43; *p* = 0.03) and enlargement of LAVI (OR = 1.68, 95% CI, 1.01–2.80; *p* = 0.04).

**TABLE 3:** Univariate binary logistic regression analysis of GLS, GCS, E/Em, LVH and LAVI in gout patients.

Decreased GLS (>-20%)		
Characteristics	OR (95%CI)	*P*
BMI (kg/m^2^)	**1.17 (1.02–1.34)**	**0.03**
HBA1C (%)	**3.74 (1.26–11.14)**	**0.02**
FPG(mmol/L)	**1.26(1.08–1.88)**	**0.03**
Hypertension	2.85 (0.64–12.74)	0.17
Diabetes	1.09 (0.30–3.96)	0.90
Tophi	2.52 (0.98–6.53)	0.06
Decreased GCS (>-30%)		
Urea (mmol/L)	**1.14 (1.00–1.31)**	**0.04**
ESR (mm/h)	**1.02 (1.00–1.03)**	**0.03**
Hypertension	1.64 (0.78–3.44)	0.19
Diabetes	1.35 (0.58–3.11)	0.49
Tophi	1.19 (0.67–2.13)	0.56
Increased E/Em (>14)		
Age(year)	**1.09(1.05–1.13)**	**<0.001**
RBC (10^12/L)	**0.49 (0.26–0.92)**	**0.03**
HGB (g/L)	**0.97 (0.95–0.99)**	**0.004**
ALB (g/L)	**0.90 (0.82–0.99)**	**0.03**
Hypertension	**5.98 (2.35–15.25)**	**<0.001**
Diabetes	2.49 (0.83–7.42)	0.10
Tophi	2.48 (0.90–6.78)	0.08
LVH*		
Age(year)	**1.08 (1.04–1.12)**	**<0.001**
Course of disease (year)	**1.12 (1.04–1.20)**	**0.002**
RBC (10^12/L)	**0.25 (0.12–0.53)**	**<0.001**
HGB (g/L)	**0.95 (0.92–0.97)**	**<0.001**
Urea (mmol/L)	**1.21 (1.05–1.40)**	**0.008**
Cys-C (mg/L)	**3.38 (1.27–8.95)**	**0.02**
β2-MG (mg/L)	**1.33 (1.03–1.72)**	**0.03**
TP (g/L)	**0.87 (0.81–0.94)**	**0.001**
ALB (g/L)	**0.74 (0.65–0.84)**	**<0.001**
**A/G**	**0.03 (0.00–0.29)**	**0.002**
ESR (mm/h)	**1.03 (1.01–1.05)**	**0.001**
Hypertension	**6.69 (2.33–19.20)**	**<0.001**
Diabetes	2.60 (0.78–8.70)	0.12
Tophi	**7.04 (1.54–32.29)**	**0.01**
Enlargement of LAVI (LAVI > 34 mL/m^2^)		
Age(year)	**1.08 (1.04–1.12)**	**<0.001**
Course of disease (year)	**1.08 (1.12–1.15)**	**0.006**
RBC (10^12/L)	**0.52(0.33–0.81)**	**0.01**
HGB (g/L)	**0.97 (0.95–0.99)**	**<0.001**
Urea(mmol/L)	**1.18(1.03–1.35)**	**0.02**
ALB (g/L)	**0.94 (0.88–1.00)**	**0.04**
Cys-C (mg/L)	**6.22 (1.92–20.10)**	**0.002**
β2-MG (mg/L)	**1.49 (1.13–1.97)**	**0.005**
LDL-C(mmol/L)	**0.59(0.40–0.87)**	**0.008**
Diabetes	0.89 (0.19–4.13)	0.88
Tophi	**14.82 (1.91–115.18)**	**0.01**

*Left ventricular hypertrophy (LVH) was diagnosed as LVMI>95 g/m^2^ in females and LVMI>115 g/m^2^ in males. Bold values: Refers to statistically significant indicators.

**TABLE 4:** Multivariate binary logistic regression analysis results of GLS, GCS, E/Em, LVH and LAVI in gout patients.

Dependent variable	Independent variable	B	OR	95% CI	*P*
Decreased GLS (>-20%)	FPG	**0.85**	**2.34**	**1.01–5.39**	**0.04**
Decreased GCS (>-30%)	Urea	**0.36**	**1.40**	**1.07–1.85**	**0.02**
Increased E/Em (>14)	Age	**0.09**	**1.09**	**1.04–1.16**	**0.001**
	Hypertension	**2.12**	**8.35**	**1.83–38.02**	**0.006**
LVH*	Urea	**0.47**	**1.59**	**1.04–2.43**	**0.03**
Enlargement of LAVI (LAVI > 34 mL/m^2^)	Urea	**0.52**	**1.68**	**1.01–2.80**	**0.04**

*Left ventricular hypertrophy (LVH) was diagnosed as LVMI>95 g/m^2^ in females and LVMI>115 g/m^2^ in males. Bold values: Refers to statistically significant indicators.

## 4 Discussion

Our findings showed that decreased GLS and GCS, LVH, and enlargement of LAVI were associated with urea levels. Previous evidence suggested that left atrial remodeling was an important value in predicting the risk and mortality of cardiovascular events, and left atrial enlargement played an important role in the development of heart failure (Welles et al., 2012; Inoue et al., 2021). Also, LAVI is a robust indicator of diastolic dysfunction independent of left ventricular loading conditions (Douglas, 2003; Singh et al., 2017). The left atrium maintains the normal function of the heart by regulating the filling process of the left ventricle and affecting the rhythm of myocardial contraction and diastole. In addition, the left atrium also has reservoir, conduit, and reinforcement functions, and is able to adaptively change its structural and mechanical properties to maintain its normal function (Pathan et al., 2017). Enlargement of the left atrium is considered to be one of the important markers of the development of heart disease, especially closely related to the development of heart failure (Ye et al., 2020). A number of studies have shown that high urea levels were associated with adverse cardiovascular outcomes, and elevated urea could predict renal hypoperfusion, a renal hemodynamic state that may be associated with reduced cardiac output (Jujo et al., 2017; Carnicelli et al., 2020). Taken all these together, our study suggested that elevated urea might be a good predictor of changes in myocardial subclinical systolic function in gout patients. In addition, urea is also considered as a risk factor for diastolic dysfunction and left ventricular hypertrophy in gout patients. This might related to the activation of a complex neurohormonal mechanism that stimulates the release of vasopressin and activates the renal sympathetic nervous system (Fang et al., 2017). Yanwei Du et al. have also demonstrated that high urea could lead to hypertrophy cardiomyocytes in mouse (Du et al., 2014). They found that the deletion of urea channel protein would not only lead to the accumulation of urea in mouse cardiomyocytes, and also lead to the increase of myocardial reactive oxygen species production and increased oxidative stress in mouse. In addition, the increase of oxidative stress from reactive oxygen species was suggested to associate with myocardial fibrosis, myocardial hypertrophy, LV remodeling, impaired myocardial contractility, and decreased cardiac function (Herrmann et al., 2012).

Our study also showed that GLS was positively correlated with FPG, and LVEF was negatively correlated with FPG. After the logistic regression analysis, we found that increased FPG was a risk factor for absolute decrease of GLS. Diabetes could result in the structural abnormalities of the heart (Zhao et al., 2020; Turkes et al., 2021). And it is an important risk factor for heart failure, suggesting that glycemic control may influence the development of heart failure (Gilbert and Krum, 2015). Elevated FPG has been shown to be prediabetic, and also a risk factor for heart failure in non-diabetic patients (Dauriz et al., 2017). Poor glycemic control in diabetic patients may cause cardiomyocyte hypertrophy, collagen deposition and cross-linking, and further lead to increased LV mass and enhanced myocardial oxidative stress, all of which may lead to impaired myocardial function (Harada et al., 2017). This is also consistent with our findings that elevated FPG might be a significant predictor of subclinical systolic dysfunction in gout patients.

It has been found that gout can cause changes in LV diastolic function, and patients with gout can have diastolic dysfunction before systolic dysfunction (Pan et al., 2014b). E/Em is a sensitive indicator of LV diastolic function, which increases as LV diastolic function decreases (Dudziak et al., 2016). Our findings demonstrated that age and hypertension were risk factors for increased E/Em, suggesting that patients with gout might have an increased risk of LV diastolic dysfunction with age and secondary hypertension. Hypertension is considered to be an important factor in the development of LV hypertrophy (Aronow, 2017). Previously, the cardiac structural change of LV hypertrophy was thought to be related with hypertension, but recent studies showed that metabolic syndrome was also associated with the development of LV remodeling (Homan et al., 2019). Several hypotheses including angiotensin II stimulation, cardiomyocyte hypertrophy, high insulin levels, and the role for the sympathetic nervous system have been proposed for the explanation of LV remodeling. Whereas it is difficult to determine which mechanisms was conclusive as these mechanisms were often jointly found in patients with gout. Hyperuricemia and elevated uric acid levels were potential risk factors for hypertension, which was a major cause of cardiovascular disease (Stewart et al., 2019). Therefore, it is necessary to monitor the chanegs of blood pressure in gout patients to reduce the risk of LV remodeling. In addition, gout was associated with an increased risk of heart failure in the elderly patients, hence early changes in cardiac function should be especially noted in elderly patients when clinical interventions were performed in patients with gout (Colantonio et al., 2020).

This study has several limitations that need to be acknowledged. Firstly, it is a single-center study, which may introduce regional bias and limit the generalizability of the research findings. Conducting multi-center or cross-regional studies would be necessary to validate the stability and applicability of the results. Secondly, the sample size is relatively small, which may affect the statistical power and reliability of the study. Further expansion of the sample size is needed to enhance the credibility and generalizability of the research. Thirdly, the majority of patients in this study are males, with a relatively small number of females included. Considering the physiological differences between genders, gender factors may interfere with the associations between cardiac ultrasound parameters and laboratory indicators. Future studies should strive for a better balance in gender distribution to explore the influence of gender on the research results. Additionally, there may be issues with data quality in this study. Although efforts have been made to ensure the accuracy of data collection and processing, measurement errors or incomplete data records may still exist. This could potentially impact the reliability and interpretation of the results. Furthermore, there are unmeasured confounding factors in our study. Apart from the potential factors we have considered, there may be other unanalyzed factors that could influence the results. This may lead to the inability to completely eliminate the possibility of confounding. Lastly, we acknowledge the potential statistical issues when conducting multiple tests. Since we have performed multiple correlation analyses, there is an increased chance of discovering chance results. Therefore, future research needs to employ more rigorous statistical methods to validate our findings.

In conclusion, our study found that elevated urea and FPG were risk factor for subclinical LV myocardial dysfunction in patients with gout, which might provide a theoretical basis for the early diagnosis and treatment of heart disease in clinical practice. While caution must be taken in generalising these findings to female patients due to potential gender differences in this study, they provide a valuable theoretical basis for the early diagnosis and treatment of heart disease in patients with gout in clinical practice (Redon et al., 2019). Besides, older patients with gout and hypertension should be close monitored to prevent the occurrence of cardiovascular events.

## Data Availability

The raw data supporting the conclusion of this article will be made available by the authors, without undue reservation.
