# Corrigendum: Analysis of risk factors for changes of left ventricular function indexes in Chinese patients with gout by echocardiography

**DOI:** 10.3389/fphys.2024.1373812

**Published:** 2024-05-14

**Authors:** Wantai Dang, Danling Luo, Jing Hu, Hui Luo, Xiaohui Xu, Jian Liu

**Affiliations:** ^1^ Department of Rheumatology and Immunology, Clinical Medical College, The First Affiliated Hospital of Chengdu Medical College, Chengdu, China; ^2^ Department of Ultrasound, Clinical Medical College, The First Affiliated Hospital of Chengdu Medical College, Chengdu, China

**Keywords:** left ventricular function, gout, echocardiography, uric acid, risk factors

In the published article, an **Author Name** was incorrectly written as Jin Hu. The correct spelling is Jing Hu. 

Jian Liu (liujiansh@126.com) is the sole **Corresponding author**.

The authors apologize for these errors and state that this does not change the scientific conclusions of the article in any way. The original article has been updated.

